# Prostatic Hypertrophy—Its Symptomatology and Diagnosis1See page 661 June Number of this Journal.

**Published:** 1906-07

**Authors:** E. A. Babler


### Prostatic Hypertrophy---Its Symptomatology and Diagnosis.[1]

[*Saint Louis Courier of Medicine, May. 1906.*]

EXPERIENCE daily corroborates the contention that the discrepancy between the true amount of prostatic hypertrophy and the severity of the clinical manifestations is often very great. A patient possessing a considerably enlarged prostate may not complain of any very annoying or severe symptoms; in some instances a slightly enlarged organ will cause symptoms out of all proportion to the amount of hypertrophy. It seems quite probable, however, that there is some relation between the part of the gland involved, the extent of the pathological changes in the neck of the bladder, the patency and resiliency of the prostatic urethra and the severity of the symptoms. Personally, I can not agree with Keyes, who compares the relation between the bladder and urethra with that between a tank and its outflow pipe, and who would lead us to believe that mere elevation of the urethral orifice plays such an important factor. To me it seems that the difficulty lies in the pathological changes which have taken place in the prostatic tis-

1. See page 661 June Number of this Journal.

sue, in the resiliency and patency of the prostatic urethra, and at the same time in the neck of the bladder. Sometime since, Chetwood called attention to the very important finding that prostatism may be due to contracture of the neck of the bladder.

German writers prefer to divide the symptoms of prostatic enlargement into three stages: In the first stage urination is difficult while the bladder is capable of emptying itself completely; in the second stage there is incomplete or complete retention; and in the third stage there is incontinence of urine with distention of the bladder. Keyes enumerates the chief symptoms under three heads:

1. Congestve symptoms, viz., priapism, nocturnal frequency of urination and prostatic neuralgia.
2. Inflammatory symptoms, viz., prostatitis, cystitis and pyelonephritis—to mention only the chief ones.
3. Obstructive symptoms, viz., retention of urine. (He believes that the ultimate cause of the symptoms of prostatic hypertrophy is congestion).

Perhaps the most constant early symptom of enlargement of the prostate is the increased frequency of urination. In some instances an inability to satisfactorily perform the act, a dribbling of urine after the act has apparently been completed, increased tenesmus, an inability to project the urine or, perhaps, an attack of acute retention, will be the first symptom to command the serious consideration of the individual. Very frequently the patient regards the gradually-appearing discomforts as natural accompaniments of his advancing years. Concerning the early clinical picture, Dr. H. G. Mudd has very fittingly said:

"In the majority of cases but little attention is given to the preliminary manifestations; the increased frequency of urination, the retention of a few drops which dribble out after the act is apparently finished. Only when bleeding, pain or obstruction seriously inconveniencing the patient, show themselves, is the matter considered of importance enough to consult the medical adviser."

The clinical picture of enlargement of the prostate is too well known to require but passing comment. The patient, usually an individual who has passed the meridian of life, at first passes his urine at more frequent intervals. He finds it necessary to urinate just as soon as he arises in the morning; gradually he is required to get up at night; he then voids his urine immediately before retiring in the evening, since the call is thereby delayed. As the months come and go he discovers that it is sometimes impossible to avoid instantly passing urine the moment the desire is felt; he can not project his urine as formerly; the flow is liable to a sudden stoppage and, though the stream is as large as formerly, still the act requires a longer time and straining is of no avail. About this time the patient notices that there is a dribbling from the urethra after the act of micturition has apparently been completed—a very annoying and distressing symptom. The last phase is the occurrence of an attack of retention. Urinary rerention is undoubtedly a most distressing condition.

### DIAGNOSIS

The diagnosis must be based upon the previous history, the findings obtained by rectal palpation, the information secured by the catheter and by examination of the urine. Some investigators believe that the cystoscope will be found useful in reaching a diagnosis of prostatic hypertrophy, but Syms says:

"The diagnosis should be made in the simplest manner possible. In a patient, 50 years of age or more, pronounced change in the act of urination should lead one to suspect prostatic obstruction. A carefully taken history of the case will put one entirely in possession of the subjective symptoms and no examination should be made beyond palpation of the prostate through the rectum and the very careful introduction of a flexible catheter into the bladder, after the patient has urinated, for the purpose of determining the amount of residual urine and the quality of the same. I can not speak too strongly against the employment of the cystoscope in these cases. I regard a cystoscopic examination of a prostatic patient who is suffering from a more or less severe cystitis as a thoroughly unwarranted procedure, and as far more dangerous than a properly performed removal of the prostate through the perineum."

In some instances a chronic prostatitis, new growths or calculi of the prostate or bladder, or strictures of the urethra, in so far as they obstruct the passage of urine, may be mistaken for prostatic hypertrophy. In chronic prostatitis the three-glass test will show typical comma filaments, pus and, perhaps, gonococci (even in the middle glass). A sarcoma or a carcinoma involving an otherwise normal prostate may be difficult to differentiate; when the gland has become irregular, nodular and of hard consistence, rectal palpation will render the diagnosis fairly clear. A calculus of the prostate usually forms in the gland, it feels hard while the other portions of the gland are soft, perhaps, abnormally so (Nitze). Tumors of the bladder should be carefully examined and tumor fragments should be searched for in the sediment. An hourglass-shaped vesical calculus may give trouble in diagnosis but the history of the case and a careful examination will clear up the obscurity. In the next issue the treatment will be discussed. —E. A. BABLER.

